# Einfluss der COVID-19-Pandemie auf die ambulante psychotherapeutische Versorgung von Kindern und Jugendlichen

**DOI:** 10.1007/s00278-022-00604-y

**Published:** 2022-06-27

**Authors:** Maria Plötner, Katja Moldt, Tina In-Albon, Julian Schmitz

**Affiliations:** 1 Abteilung Klinische Kinder- und Jugendpsychologie, Universität Leipzig, Neumarkt 9–19, 04109 Leipzig, Deutschland; 2 Klinische Psychologie und Psychotherapie des Kindes- und Jugendalters, Universität Koblenz-Landau, Campus Landau, Landau, Deutschland

**Keywords:** Psychische Störungen, Coronapandemie, Wartezeiten, Psychotherapie, PsychotherapeutInnen, Mental disorders, Corona pandemic, Waiting times, Psychotherapy, Psychotherapists

## Abstract

**Hintergrund:**

Seit Beginn der COVID-19-Pandemie mehren sich Befunde zu ihrem negativen Einfluss auf die psychische Gesundheit von Kindern und Jugendlichen. Bisher ist jedoch wenig darüber bekannt, ob und wie sich dies auf die psychotherapeutische Versorgung von Kindern und Jugendlichen niederschlägt.

**Ziel der Arbeit:**

Die psychische Situation von Kindern und Jugendlichen sowie ihre psychotherapeutische Versorgung seit Beginn der COVID-19-Pandemie sollen aus Sicht von Kinder- und JugendlichenpsychotherapeutInnen (KJP) erfasst werden.

**Material und Methoden:**

Es wurden 324 KJP aus Deutschland in einer Online-Umfrage gebeten, die letzten 6 Monate mit einem 6‑monatigen Zeitraum vor 2 Jahren zu vergleichen. Fünf- und 7‑stufige Likert-Skalen, Fragen mit Mehr- und Einfachauswahl sowie numerische und ein freies Antwortformat wurden verwendet.

**Ergebnisse:**

Seit Pandemiebeginn haben sich die Wartezeiten nahezu verdoppelt. Es werden mehr Behandlungsstunden angeboten, v. a. mehr Erstgespräche durchgeführt. Therapieverlängerungen kommen häufiger, -abbrüche seltener vor. Bei der Hälfte der PatientInnen ist eine pandemieassoziierte Symptomverschlechterung aufgetreten. Alle erfragten psychischen Störungen treten z. T. deutlich häufiger auf (v. a. Depressionen, Angststörungen, Medienabhängigkeit, Schlaf‑, Anpassungs‑, Zwangs- und Essstörungen). Es erfolgen mehr Telefon- und Videositzungen als vor der Pandemie. Die Zusammenarbeit mit Eltern hat sich verstärkt, die mit dem interdisziplinären Netzwerk verringert.

**Diskussion:**

Die Pandemie hat einen deutlichen Einfluss auf die psychische Verfassung und die psychotherapeutische Versorgung von Kindern und Jugendlichen in Deutschland. Eine Anpassung des Versorgungssystems an den gestiegenen Bedarf wird vorgeschlagen, um mögliche Folgeschäden der Pandemie zu begrenzen.

**Zusatzmaterial online:**

Die Online-Version dieses Beitrags (10.1007/s00278-022-00604-y) enthält die detaillierten Fragen des Fragebogens.

## Abstract (en)

**Background:**

Since the start of the COVID-19 pandemic there is a growing body of evidence showing its negative impact on the mental health of children and adolescents. However, little is known about whether and how this impacts the psychotherapeutic care for children and adolescents.

**Objective:**

The mental health of children and adolescents as well as their psychotherapeutic care since the onset of the COVID-19 pandemic were investigated from the perspective of child and adolescent psychotherapists (CAPs).

**Material and methods:**

We conducted an online survey with 324 CAPs from all over Germany. Participants were asked to compare the last 6 months with a 6-month period 2 years ago. The survey used 5‑point and 7‑point Likert scales, questions with single and multiple choice options as well as numeric and open answer formats.

**Results:**

Since the beginning of the pandemic waiting times have nearly doubled. CAPs offered more treatment sessions and, in particular, more initial consultations were carried out. Therapy extensions were more frequent and therapy terminations occurred less often. In about half of the patients, a pandemic-associated aggravation of symptoms occurred. All of the reported mental disorders occurred more often, in particular depression, anxiety disorders, media addiction, sleep, adjustment, obsessive-compulsive and eating disorders. More telephone and video sessions were carried out than before the pandemic. The pandemic resulted in a closer cooperation with parents but a decreased cooperation with other disciplines.

**Conclusion:**

The pandemic had a significant impact on the mental health of and the psychotherapeutic care for children and adolescents in Germany. An adaptation of the mental health system to the increased need for therapy is proposed in order to limit future mental and economic harm.

„Die Nerven liegen bei Familien blank.“ – So fasst eine Teilnehmerin der vorliegenden Studie zusammen, welche Auswirkungen der COVID-19-Pandemie sie in ihrer Arbeit als Kinder- und Jugendlichenpsychotherapeutin feststellen konnte. Seit Beginn der Pandemie mehren sich Befunde zum negativen Einfluss der Coronakrise auf die psychische Gesundheit. Dennoch ist noch wenig darüber bekannt, ob und wie sich dieser Einfluss auf die psychotherapeutische Versorgung von Kindern und Jugendlichen niederschlägt.

## Hintergrund und Fragestellung

Das Kindes- und Jugendalter stellt eine besonders vulnerable Phase für die Entwicklung psychischer Störungen dar und ist daher für die gesamte weitere Entwicklung von zentraler Bedeutung (Brakemeier et al. 2020). Laut epidemiologischen Studien weist jede bzw. jeder 6. Heranwachsende in Deutschland Anzeichen für psychische Störungen auf (Barkmann und Schulte-Markwort 2012). Diese manifestieren sich oftmals frühzeitig, die Hälfte vor dem 14. Lebensjahr (Lambert et al. 2013). Das Vorliegen psychischer Auffälligkeiten in Kindheit und Jugend gilt als Risikofaktor für die Entwicklung psychischer Störungen im späteren Lebensalter (Kim-Cohen et al. 2003). Psychische Störungen im Kindes- und Jugendalter sollten daher frühzeitig erkannt und behandelt werden.

### Einfluss der Coronapandemie auf die psychische Gesundheit von Kindern und Jugendlichen

Die COVID-19-Pandemie der vergangenen 2 Jahre stellte einen Belastungstest für Menschen aller Altersgruppen dar. Während zu Pandemiebeginn der Fokus des Gesundheitssystems vorrangig auf der Infektionsvermeidung und der Gewährleistung der medizinischen Versorgung lag, rückte nach und nach auch die psychische Gesundheit in den öffentlichen Fokus. Fachleute hoben die Pandemie und die einhergehenden Maßnahmen zum Schutz der Bevölkerung als massiven Stressor für die psychische Gesundheit hervor (Brakemeier et al. 2020; Gruber et al. 2020). Dabei schien sich abzubilden, dass Kinder und Jugendliche auf den pandemieassoziierten Stress besonders stark reagieren (COVID-19 Mental Disorders Collaborators 2021; Singh et al. 2020). Während ihr eigenes Risiko für schwere COVID-19-Verläufe geringer zu sein scheint als bei Erwachsenen (Ludvigsson 2020), sorgen sie sich um die Gesundheit ihrer Angehörigen (Sarkadi et al. 2021). In der JuCo-Studie berichten Kinder und Jugendliche seit Pandemiebeginn mehr Stress in der Schule, während gleichzeitig weniger Ausgleich durch Freizeitmöglichkeiten oder Kontakt zu Peers möglich ist (Andresen et al. 2020). Die Lebensqualität der Kinder und Jugendlichen hat sich in der ersten Phase der Pandemie reduziert (Ravens-Sieberer et al. 2020); hinzu kommt ein Gefühl des Nicht-gehört- und Nicht-ernst-genommen-Werdens (Andresen et al. 2020). Kinder und Jugendliche werden durch die pandemiebedingten Einschränkungen jedoch nicht nur direkt, sondern auch indirekt, über vermittelnde Faktoren, belastet. Besonders der Wegfall unterstützender privater und institutioneller Strukturen wirkt sich negativ auf Familien aus (Knabe et al. 2021). Während der Lockdowns berichteten Eltern ein erhöhtes Stresserleben durch die Mehrfachbelastung von Kinderbetreuung, Homeschooling und Homeoffice, was das Risiko für schädigendes Erziehungsverhalten erhöhte (Clemens et al. 2021). Elterliches Stresserleben in der Pandemie sagte verringertes kindliches Wohlbefinden und Problemverhalten vorher (Essler et al. 2021).

Empirische Studien aus der ganzen Welt zeigen zudem, dass die Pandemie die Entstehung psychopathologischer Symptomatik befördert. So zeigte sich ein Anstieg klinisch relevanter psychopathologischer Symptome in der Allgemeinbevölkerung (für eine Metaanalyse, siehe Luo, Guo, Yu et al., 2020). Psychisch erkrankte Menschen scheinen besonders von den Auswirkungen der Pandemie betroffen zu sein (De Hert et al. 2021): Etwa 20 % der erwachsenen PatientInnen mit psychischen Störungen berichten eine Verschlechterung ihrer Symptome seit Pandemiebeginn (Zhou et al. 2020). Auch bei Kindern und Jugendlichen kann ein Anstieg psychopathologischer Symptomatik verzeichnet werden: Daten der COPSY-Studie zur psychischen Gesundheit von Kindern und Jugendlichen aus Deutschland zeigen, dass sich das Risiko für psychische Auffälligkeiten seit Beginn der COVID-19-Pandemie um das 1,5Fache erhöht hat (Ravens-Sieberer et al. 2020, 2021). Symptome der Hyperaktivität, emotionale Probleme, Verhaltensauffälligkeiten sowie psychosomatische Beschwerden haben im ersten Pandemiejahr zugenommen. Eine Metaanalyse zeigt sogar eine Verdopplung klinisch auffälliger Depressions- und Angstsymptome bei Kindern und Jugendlichen seit Pandemiebeginn (Racine et al. 2021). Es ist jedoch wichtig zu beachten, dass die zitierten Studien auf Fragebogenerhebungen mit Kindern und Jugendlichen selbst oder ihren Bezugspersonen beruhen. Diese Studien können wichtige Hinweise darüber geben, ob Betroffene oder ihre Eltern Symptome beobachten, die klinische Relevanz aufweisen und einer professionellen Abklärung bedürfen. Sie können jedoch keine Auskunft über das Vorliegen psychischer *Störungen* liefern, deren Diagnosen nur von Fachpersonal nach ausführlichen Diagnosegesprächen und -erhebungen vergeben werden können. Im Fremdbericht der Eltern können zudem eigene pandemiebedingte Belastungen das Erleben und Einschätzen der kindlichen Belastungen prägen. In der vorliegenden Studie soll daher die Einschätzung von Kinder- und JugendlichenpsychotherapeutInnen (KJP) erhoben werden.

### Psychotherapie während der Pandemie

Mit der Zunahme psychischer Belastungen war gleichzeitig die Durchführung von Psychotherapien aufgrund der geltenden Beschränkungen erschwert. Die pandemiebedingten Beschränkungen erforderten zeitweise eine Anpassung des Therapiesettings. Viele Psychotherapien mit Erwachsenen wurden seit Pandemiebeginn in den digitalen Raum verlagert (Eichenberg 2020). Obwohl digitale Hilfsangebote auch bei Kindern und Jugendlichen bereits in der Vergangenheit angewandt und als effektiv bewertet wurden (Martinelli et al. 2020; Racine et al. 2021), sind niedergelassene PsychotherapeutInnen gegenüber Video- und Teletherapie mit Kindern bisher eher skeptisch (Rabe-Menssen et al. 2020).

## Ziel der Studie

Die vorliegende Studie hat zum Ziel, die psychische Situation von Kindern und Jugendlichen sowie ihre psychotherapeutische Versorgung seit Beginn der COVID-19-Pandemie aus Sicht von KJP zu erfassen. Folgende Hypothesen wurden aufgestellt:

- Aufgrund des gestiegenen Risikos für psychische Auffälligkeiten bei Kindern und Jugendlichen (Ravens-Sieberer et al. 2020, 2021) und des daraus wahrscheinlich resultierenden erhöhten Behandlungsbedarfs wird eine Erhöhung der Wartezeiten auf einen Therapieplatz erwartet.
- Es wird vermutet, dass KJP ihre Therapieangebote seit Pandemiebeginn an den gestiegenen Bedarf angepasst haben (z. B. mehr Therapiestunden anbieten).
- Da die Pandemie insbesondere bereits psychisch erkrankte Menschen zu beeinträchtigen scheint (De Hert et al. 2021), werden eine Veränderung der Therapieverläufe (z. B. häufigere Therapieverlängerungen) sowie eine Zunahme von Wiederanfragen vorbehandelter PatientInnen angenommen. Analog zu vorliegenden Befunden zu pandemieassoziierten Symptomaggravationen bei erwachsenen PatientInnen (Zhou et al. 2020) werden Symptomverschlechterungen bei den PatientInnen der befragten KJP für wahrscheinlich gehalten.
- Entsprechend den Befunden zu erhöhten Angststörungs- und Depressionsraten (Racine et al. 2021) wird erwartet, dass KJP das Auftreten der entsprechenden Störungen als häufiger einschätzen als vor der Pandemie.
- Aufgrund der pandemiebedingten Kontaktbeschränkungen wird, analog zu den Entwicklungen in der therapeutischen Arbeit mit Erwachsenen (Eichenberg 2020), mit einer Zunahme von Tele- und digitalen Therapieformaten sowie einer Verringerung der interdisziplinären Zusammenarbeit gerechnet.

## Studiendesign und Untersuchungsmethoden

### Rekrutierung

Die Rekrutierung erfolgte über die E‑Mail-Verteiler deutscher Psychotherapeutenkammern und -verbände sowie über das postalische Anschreiben niedergelassener KJP.

### Stichprobe

Im Befragungszeitraum haben 487 Teilnehmende die Umfrage begonnen, von denen 328 diese abschlossen. Vier Teilnehmende wurden von der Datenanalyse ausgeschlossen, da sie weniger als 2 Jahre Berufserfahrung aufwiesen, wodurch ein Vergleich zum Vor-Pandemie-Zeitraum nicht möglich war (*n* = 3) oder die Bearbeitungszeit des Fragebogens unrealistisch kurz erschien (*n* = 1). Die finale Stichprobe umfasst 324 KJP (258 weiblich, 66 männlich) mit einem durchschnittlichen Alter von 47,71 Jahren (Standardabweichung [SD] ± 9,74 Jahre, Minimum [Min.] 27 Jahre, Maximum [Max.] 75 Jahre). Fast die Hälfte der Befragten arbeitet in Großstädten (*n* = 160; 49,38 %), mehr als ein Drittel in kleineren bzw. mittleren Städten (*n* = 139; 42,9 %) und weniger als ein Zehntel im ländlichen Raum (*n* = 27; 8,33 %). Es waren Teilnehmende aus allen 16 Bundesländern vertreten. Die durchschnittliche Zahl der Berufsjahre betrug 12,14 (SD ± 8,17). In Tab. 1 ist die therapeutische Arbeitssituation der Befragten dargestellt.

**Table 1:** 

Arbeitssituation	Anzahl (*n*)	Anteil (%)
*Praxisart*		
Vertragspsychotherapeutische Praxis	272	83,95
Privatpraxis	28	8,64
Andere Institution der ambulanten psychotherapeutischen Versorgung	24	7,41
*Zulassung*		
Volle Zulassung (ganzer Kassensitz)	129	39,81
Teilzulassung (halber Kassensitz)	140	43,21
Angestelltenverhältnis	35	10,80
Keine Angabe	20	6,17
*Approbation*		
Einzelapprobation als KJP	285	87,96
Psychologische/r PsychotherapeutIn mit Abrechnungsgenehmigung für Kinder und Jugendliche	23	7,10
Doppelapprobation als psychologische PsychotherapeutIn und KJP	16	4,94

Es wurden nur Teilnehmende in die Studie eingeschlossen, die den Fragebogen vollständig und während der offiziellen Datenerhebungszeit ausgefüllt hatten.

### Fragebogen und Datenerhebung

Der Fragebogen wurde mit Unterstützung einer 6‑köpfigen ExpertInnengruppe aus VertreterInnen der Wissenschaft und der ambulanten psychotherapeutischen Versorgung von Kindern und Jugendlichen entwickelt. Die Befragung erfolgte vom 10.05.2021 bis 31.07.2021 im Online-Fragebogenportal „Unipark“ (https://www.unipark.de/). Die durchschnittliche Bearbeitungsdauer ohne Unterbrechung betrug 14 min 56 s. In 23 Fragen wurden Teilnehmende gebeten, die psychische Situation von Kindern und Jugendlichen sowie die psychotherapeutische Versorgungssituation im Zeitraum der letzten 6 Monate mit einem 6‑monatigen Zeitraum vor 2 Jahren zu vergleichen. Hierfür wurden Fragen mit Mehrfach- und Einfachauswahl verwendet, sowie einfache Ratingauswahlen mithilfe einer 7‑stufigen Likert-Skala von „sehr stark verringert“ bis „sehr stark erhöht“ bzw. „sehr viel seltener“ bis „sehr viel häufiger“ oder einer 5‑stufigen Likert-Skala von „sehr häufig“ bis „gar nicht“. Mithilfe numerischer Antwortformate wurden die Zahl der Jahre (z. B. Berufsjahre) oder Wochen (z. B. durchschnittliche Wartezeit) erfasst. Am Ende des Fragebogens gab es ein freies Antwortformat. Die detaillierten Fragen des Fragebogens können im Zusatzmaterial online eingesehen werden.

Die Studie erhielt am 04.05.2021 ein positives Votum des Ethikbeirates der Universität Leipzig (AZ 2021.04.23_eb_84).

### Statistische Analyse

Zur Auswertung der numerischen Fragen wurden einfache *t*-Tests und für die Auswertung der Fragen mit Likert-Skalen Wilcoxon-Tests für einzelne Stichproben verwendet. Es wurden Holm-Bonferroni-Korrekturen für alle Analysen innerhalb eines Fragenkomplexes angewandt.

## Ergebnisse

### Wartezeiten und angebotene Behandlungsstunden

Die Teilnehmenden berichteten, dass PatientInnen bei ihnen aktuell signifikant länger auf einen Erstgesprächstermin oder Therapieplatz warten als im Vergleichszeitraum vor der Pandemie (Tab. 2).

#### Merke.

Die Wartezeiten für PatientInnen haben sich während der COVID-19-Pandemie nahezu verdoppelt.

**Table 2:** 

	Wartezeiten (Wochen)		
	M	SD	Signifikanztestung
*Erstgespräch*			
Vor 2 Jahren	5,8	± 7,1	Differenz > 0; *t* (323) = 10,958, *p* < 0,0001
Letzte 6 Monate	10,2	± 11,7	
*Therapieplatz*			
Vor 2 Jahren	14,4	± 13,6	Differenz > 0; *t* (323) = 13,727, *p* < 0,0001
Letzte 6 Monate	25,3	± 22,6	

*M* Mittelwert, *SD* Standardabweichung

Zudem berichteten 66 % der Teilnehmenden, ihre Behandlungsstunden seit Pandemiebeginn leicht bis sehr stark erhöht zu haben, bei 23 % seien sie gleichgeblieben, und 11 % hätten sie leicht bis sehr stark verringert (signifikante durchschnittliche Erhöhung, *Z* (324) = 10,69, *p* < 0,0001, *r* = 0,59). In der anschließenden Mehrfachauswahl möglicher Einflussfaktoren auf die Behandlungsstunden wurde bei einer Erhöhung der Behandlungsstunden am häufigsten ein gestiegenes Anfrageaufkommen genannt (*n* = 183; 85,5 %). Befragte, die ihre Stunden verringert hatten, nannten Infektionsschutzmaßnahmen (*n* = 23; 62,2 %) sowie das Beschulen der eigenen Kinder (*n* = 22; 59,5 %) als häufigste Faktoren.

#### Merke.

Die Mehrzahl der TherapeutInnen hat ihre Behandlungsstunden während der COVID-19-Pandemie erhöht.

### Therapieangebot und -verläufe

**Figure 1:**
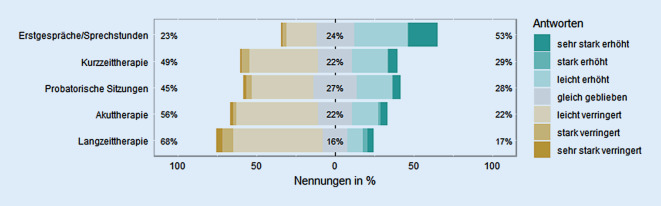

Die Befragten gaben an, im Vergleich zum Vorpandemiezeitraum habe sich die Zahl der durchgeführten Erstgesprächen signifikant erhöht, *Z* (324) = 7,19, *p* < 0,0001, *r* = 0,4, die der probatorischen und Therapiesitzungen signifikant verringert, alle *Z*s (324) > 2,68, alle *p*s < 0,01, alle *r*s ≥ 0,15; Abb. 1.

**Figure 2:**
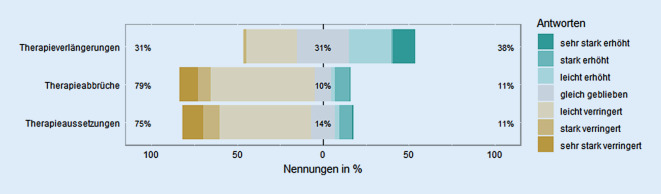

Begonnene Therapien wurden seit dem Pandemiebeginn signifikant häufiger verlängert, *Z* (324) = 3,75, *p* < 0,001, *r* = 0,21, und seltener abgebrochen, *Z* (324) = 9,99, *p* < 0,0001, *r* = 0,56, oder ausgesetzt, *Z* (324) = 9,96, *p* < 0,0001, *r* = 0,55; Abb. 2.

### Wiederanfragen und Symptomverstärkung

Befragt danach, wie viele der Neuanfragen für ein Erstgespräch bzw. einen Therapieplatz durch bereits behandelte Patienten getätigt wurden, zeigte sich eine signifikante Erhöhung im Vergleich zur Zeit vor der Pandemie. Während vor 2 Jahren noch 9,53 % (SD ± 10,05 %) der Neuanfragen durch bereits behandelte PatientInnen erfolgten, betrug der Anteil in den letzten 6 Monaten 17,16 % (SD ± 16,42 %; Differenz > 0; *t* (223) = 10,197, *p* < 0,0001).

**Figure 3:**
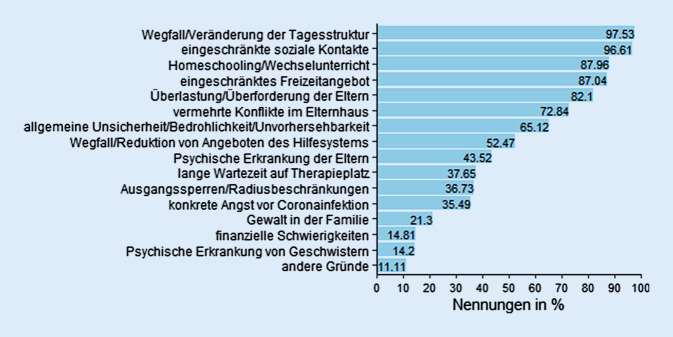

Die befragten PsychotherapeutInnen gaben zudem an, dass bei durchschnittlich 55,2 % ihrer PatientInnen in den letzten 6 Monaten eine pandemieassoziierte Verschlechterung bezüglich der Symptomzahl oder -schwere aufgetreten sei, SD ± 26,2 %; Mittelwert > 0; *t* (323) = 37,946, *p* < 0,0001. Die Gründe für die pandemieassoziierten Verschlechterungen sind in Abb. 3 dargestellt.

#### Merke.

Bei der Hälfte der PatientInnen hat sich die Symptomatik während der COVID-19-Pandemie verschlechtert.

### Häufigkeit psychischer Störungen

**Figure 4:**
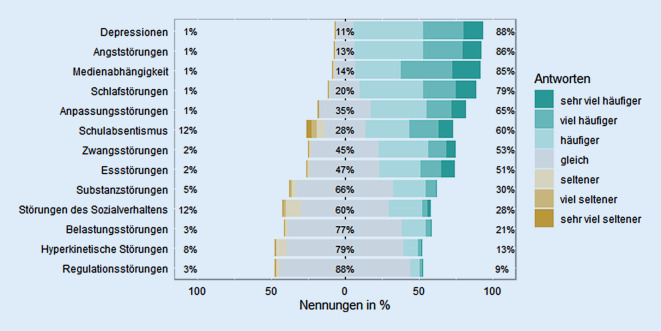

Befragt danach, wie sich bestimmte Störungsbilder in den letzten 6 Monaten im Vergleich zur Vorpandemiesituation vor 2 Jahren verändert haben, gaben die Befragten eine signifikante Zunahme aller genannten psychischen Störungen an, alle *Z*s (324) > 2,16, alle *p*s < 0,05, Abb. 4. Für die Zunahme von Depressionen, Angststörungen und Medienabhängigkeit ergeben sich sehr große Effekte (alle *r*s > 0,8), für Schlaf‑, Anpassungs‑, Zwangs- und Essstörungen große Effekte (alle *r*s = 0,6–0,8) und für Schulabsentismus ein moderater Effekt (*r* = 0,45). Die Zunahmen von Substanz‑, Belastungs-, hyperkinetischen und Regulationsstörungen sowie Störungen des Sozialverhaltens sind als klein bis sehr klein einzustufen (alle *r*s = 0,12–0,38).

#### Merke.

Psychische Störungen treten seit Beginn der COVID-19-Pandemie häufiger auf.

### Therapieformate und interdisziplinäre Zusammenarbeit

**Figure 5:**
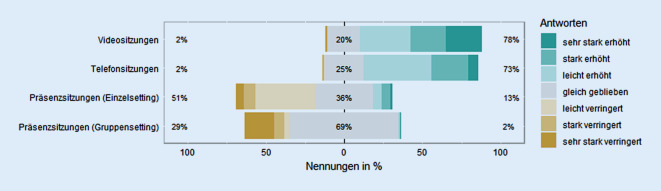

Die Befragten gaben an, das Durchführen von Video- (*Z* (324) = 13,29, *p* < 0,0001, *r* = 0,74) und Telefonsitzungen (*Z* (324) = 13,19, *p* < 0,0001, *r* = 0,73) mit PatientInnen habe sich seit Pandemiebeginn signifikant erhöht, während Präsenzsitzungen mit EinzelpatientInnen (*Z* (324) = 6,64, *p* < 0,0001, *r* = 0,37) und Gruppen verringert worden seien (*Z* (324) = 8,31, *p* < 0,0001, *r* = 0,46; Abb. 5).

**Figure 6:**
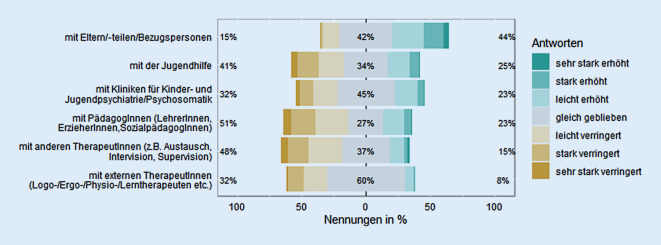

Während sich die therapeutische Zusammenarbeit mit den Eltern seit Pandemiebeginn erhöhte, *Z* (324) = 7,8, *p* < 0,0001, *r* = 0,43, hat sich die Zusammenarbeit mit externen Personen des interdisziplinären Netzwerks verringert, alle *Z*s (324) > 3,08, alle *p*s < 0,01, alle *r*s ≥ 0,17; Abb. 6.

#### Merke.

Die interdisziplinäre Zusammenarbeit zwischen den beteiligten Institutionen ist seit Beginn der COVID-19-Pandemie erschwert.

### Freies Antwortformat

Es nutzten 89 % der Befragten das freie Antwortformat, um am Ende der Befragung weitere Veränderungen, die sie seit Pandemiebeginn in ihrer therapeutischen Arbeit beobachtet hätten, hinzuzufügen. Die Aussagen ließen sich 3 übergeordneten Kategorien zuordnen: negative Auswirkungen der Pandemie, positive Auswirkungen der Pandemie und persönliche Belastungen der TherapeutInnen (Tab. 3).

**Table 3:** 

Kategorie	Beispiele	Häufigkeit (%)
Negative Auswirkungen der Coronapandemie	Zunehmende Komorbiditäten, allgemeine Unsicherheit, familiäre Konflikte, mehr Anfragen, steigender Medienkonsum	76,17
Positive Auswirkungen der Coronapandemie	Profitieren einiger Personengruppen von Homeschooling/kleineren Klassen, erhöhte Zuverlässigkeit bezüglich Erscheinen zur Therapie, Entwicklung von Rücksichtnahme	12,63
Eigene Belastungen der TherapeutInnen	Wegfall eigener Ausgleichs- und Entlastungsmöglichkeiten, Wegfall von kollegialem Austausch, vermehrte und drängendere Anfragen, Aufwand der Hygienemaßnahmen	8,96
Anderes	Umstieg auf Videosprechstunden	3,26

#### Merke.

TherapeutInnen fühlen sich durch die COVID-19-Pandemie selbst belastet.

## Diskussion

In der vorliegenden Studie wurden 324 KJP mithilfe einer Online-Umfrage zur psychischen Situation von Kindern und Jugendlichen sowie ihrer psychotherapeutischen Versorgungssituation befragt. Die Befragten berichten im Vergleich zur Zeit vor der COVID-19-Pandemie eine deutliche Erhöhung der Wartezeiten. Gleichzeitig werden mehr Behandlungsstunden angeboten. Zudem werden mehr Erstgespräche durchgeführt, dafür weniger Therapien begonnen. Therapieverlängerungen kommen häufiger, Therapieabbrüche und -aussetzen seltener vor. Es wird berichtet, der Anteil vorbehandelter PatientInnen unter den Neuanfragen sei gestiegen und bei ca. der Hälfte aller PatientInnen sei eine Verschlechterung bezüglich der Symptomzahl/-schwere aufgetreten. Die Befragten schätzen ein, dass alle erfragten psychischen Störungen z. T. deutlich häufiger auftreten (v. a. Depressionen, Angststörungen, Medienabhängigkeit, Schlaf‑, Anpassungs‑, Zwangs- und Essstörungen). Gleichzeitig gibt es weniger Präsenz-, mehr Telefon- und Videotherapiesitzungen als vor der Pandemie. Die Zusammenarbeit mit Eltern hat sich erhöht, die mit dem interdisziplinären Netzwerk verringert.

Die Befragten nehmen neben negativen auch positive Auswirkungen der Pandemie wahr und benennen eigene Belastungen.

### Längere Wartezeiten, mehr Bedarf und Anpassung der therapeutischen Versorgung

Die nahezu verdoppelten Wartezeiten bei einer gleichzeitigen Steigerung der angebotenen Behandlungsstunden von TherapeutInnenseite deuten darauf hin, dass mehr PatientInnenanfragen als vor Pandemiebeginn getätigt werden. Dies bestätigt die oben aufgestellten Hypothesen. Mehr Kinder, Jugendliche und ihre Bezugspersonen suchen aktuell psychotherapeutische Hilfe auf. Dies ist vor dem Hintergrund eines gestiegenen Risikos für psychische Auffälligkeiten nicht verwunderlich (Ravens-Sieberer et al. 2021). In ihren Freifeldantworten berichteten einzelne Teilnehmende neben einer erhöhten Zahl von Anfragen auch eine veränderte Qualität: Die Anfragen seien verzweifelter, drängender geworden. Die befragten KJP scheinen den erhöhten Anfragen mit einer Erhöhung der Behandlungsstunden und einem erhöhten Durchführen von Erstgesprächen zu begegnen.

### Negative Effekte auf psychisch erkrankte Kinder und Jugendliche

#### Symptomverschlechterung und Rückfälle

Die negativen Effekte der Pandemie v. a. auf bereits psychisch erkrankte Menschen wurden schon diskutiert (De Hert et al. 2021). Brakemeier et al. (2020) weisen darauf hin, dass die Pandemie als Stressor bei einem gleichzeitigen Wegfall von Schutzfaktoren, wie den Möglichkeiten zum Freizeitausgleich, einen besonders negativen Einfluss auf psychisch erkrankte Menschen habe. Die erhobenen Daten deuten darauf hin, dass dies auch auf psychisch erkrankte Kinder und Jugendliche zutrifft: Unter den Neuanfragen ist der Anteil der PatientInnen gestiegen, die bei den Befragten bereits einmal in Behandlung waren. Dies könnte auf eine erhöhte „Rückfallquote“ bereits psychotherapeutisch behandelter Heranwachsender hinweisen. Zudem zeigte sich bei nahezu der Hälfte der PatientInnen eine Verschlechterung bezüglich der Schwere oder Zahl der Symptome. Ähnliches berichteten erwachsene PatientInnen seit Pandemiebeginn (Zhou et al. 2020). Diese Aggravationen könnten zu den häufigeren Therapieverlängerungen beitragen und ein Grund dafür sein, dass seltener neue Therapien begonnen werden können.

#### Zunahme psychischer Störungen

Laut Einschätzung der befragten KJP begegneten ihnen in ihren Praxen die meisten psychischen Störungen häufiger als vor der Pandemie, allen voran internalisierende Störungen wie Depressionen und Angststörungen mit sehr großen Effekten. Dies deckt sich mit dem von Kindern und Jugendlichen bzw. ihren Eltern wahrgenommenen Anstieg internalisierender Symptomatik (Ravens-Sieberer et al. 2020, 2021) sowie mit dem Anstieg von Depressions- und Angststörungsdiagnosen in Kinderarztpraxen seit Pandemiebeginn (Kostev et al. 2021). Auch der seit Pandemiebeginn befürchtete Anstieg von Suchterkrankungen (Brakemeier et al. 2020), insbesondere der Mediensucht, zeigte sich in den vorgestellten Ergebnissen. Seit Pandemiebeginn ist die tägliche Online-Zeit Jugendlicher gestiegen (Medienpädagogischer Forschungsverbund Südwest 2021). Die Daten der vorliegenden Studie deuten darauf hin, dass dieser angestiegene Medienkonsum bei einigen Heranwachsenden mit Suchtverhalten einhergeht. Große Zunahmeeffekte sind auch bei Schlaf‑, Anpassungs‑, Zwangs- und Essstörungen zu verzeichnen. Die von den befragten KJP wahrgenommenen Zuwächse psychischer Störungen könnten durch eine absolute Zunahme einzelner Störungen, zunehmende Komorbiditäten oder eine erhöhte Wahrnehmung psychischer Störungen durch KJP aufgrund der Anpassung der Erstgespräche an den gestiegenen Bedarf begründet sein. Epidemiologische Studien sind notwendig, um diese Hypothesen zu untersuchen.

### Psychotherapie in Zeiten der Pandemie

Trotz bisheriger Skepsis niedergelassener KJP gegenüber Ferntherapien mit Kindern (Rabe-Menssen et al. 2020) wurden, ähnlich wie im Erwachsenenbereich (Eichenberg 2020), auch die Psychotherapien mit Kindern und Jugendlichen seit Pandemiebeginn vermehrt in Form der Tele- und Videokommunikation durchgeführt. Zwar beklagen die Behandelnden interpersonelle und technische Herausforderungen der pandemiebedingten Umstellung (McBeath et al. 2020). Doch bisherige Befunde sprechen dafür, dass die Effektivität von Ferntherapie der der Präsenztherapie nahekommt (Martinelli et al. 2020; Slone et al. 2012), und dass diese eine gute Alternative während der Pandemie bot, um PatientInnenkontakte aufrechtzuerhalten. Dies zeigt, dass das therapeutische System flexibel auf wechselnde Einschränkungen reagieren kann.

Eine mögliche positive Entwicklung ist ein erhöhtes Commitment zur Therapie. Therapien werden aktuell seltener abgebrochen; die Zusammenarbeit mit den Eltern hat sich intensiviert. In den Freifeldantworten berichteten TherapeutInnen wiederkehrend von einer erhöhten Motivation zur Teilnahme an den Sitzungen von PatientInnenseite und flexibleren zeitlichen Gestaltungsmöglichkeiten aufgrund von Online-Sitzungen.

Eine fragwürdige Entwicklung stellt die verringerte interdisziplinäre Vernetzung dar. Anders als die vornehmlich im Einzelsetting arbeitende Psychotherapie für Erwachsene zeichnet sich der Kinder- und Jugendbereich durch ein multiprofessionelles therapeutisches Arbeiten aus. Ob und wie sich die nun vorliegende pandemiebedingte Entnetzung auf den Therapieerfolg sowie das Belastungsempfinden der behandelnden KJP niederschlägt, muss zukünftig untersucht werden.

### Limitationen der Studie

Die Umfrage stützt sich auf die subjektive Einschätzung behandelnder KJP, die auch durch ihre eigene Belastungen während der Pandemie oder mögliche Rückschaufehler geprägt sein kann. Epidemiologische Studien sollten den aktuellen Wissensstand zukünftig noch ergänzen. Der Fragebogen wurde eigens für diese Studie entwickelt und sollte weiterführend auf seine psychometrischen Eigenschaften überprüft werden.

## Schlussfolgerung

### Versorgungssystem am Limit

Es scheint, als könne der gestiegene Bedarf an psychotherapeutischer Versorgung durch die bestehenden ambulanten Strukturen nicht ausreichend und zeitnah gedeckt werden. In dieser Studie zeigen sich aktuell Wartezeiten auf einen Therapieplatz von etwa einem halben Jahr, was, die Lebensspanne eines Kindes betrachtet, als ein kritisch langer Zeitraum zu bewerten ist. Bereits vor der Pandemie gab es immer wieder Kritik an der Unterversorgung psychischer Erkrankung von Kindern und Jugendlichen, begründet in Defiziten des Versorgungssystems (Karow et al. 2013). Nun haben sich die Wartezeiten für Erstgespräche und Therapieplätze nahezu verdoppelt. Eine zeitnahe Behandlung ist jedoch essenziell, um Chronifizierungen und Komorbiditäten vorzubeugen (Patton et al. 2014). In der vorliegenden Studie zeigte sich besonders eine Zunahme belastungsassoziierter Störungen, die grundsätzlich gut behandelbar sind (z. B. In-Albon und Schneider 2007). Auf Grundlage der vorliegenden Befunde kann vorgeschlagen werden, dass in der aktuellen Krisensituation eine Anpassung des Versorgungssystems an den gestiegenen Bedarf erfolgen sollte, z. B. in Form einer Bereitstellung vermehrter Therapiekapazitäten für Kinder und Jugendliche. Hierbei bedarf es zukünftig einer regelmäßigen Kontrolle des Verhältnisses von Therapiebedarf und -angebot, um den notwendigen zeitlichen Umfang der Maßnahmen abzuschätzen sowie gesundheitliche und volkswirtschaftliche Folgeschäden der Pandemie zu begrenzen.

### TherapeutInnen am Limit?

Die beschränkten Versorgungsmöglichkeiten haben nicht nur einen Einfluss auf die PatientInnen, sondern auch auf die, die sie aus Kapazitätsgründen nicht behandeln können. Die Studie gibt erste Hinweise darauf, welchen eigenen Belastungen TherapeutInnen während der Pandemiezeit unterliegen. Die Befragten versuchen, den gestiegenen Bedarf durch eine Aufstockung der Behandlungsstunden zu decken, doch dies ist nur begrenzt möglich. Eine Teilnehmerin beschreibt eindrücklich, wie ihr die lange Warteliste „langsam die Luft zum Atmen“ nehme. Der kollegiale Austausch durch Super- oder Intervision hat sich in der Pandemiezeit verringert, und der Mehraufwand durch Infektionsschutzmaßnahmen beeinträchtigt die therapeutische Arbeit. Im freien Antwortformat werden immer wieder fehlende Möglichkeiten des Freizeitausgleichs genannt, wodurch die Psychohygiene als TherapeutIn erschwert sei. Manche KollegInnen sorgen sich daher um das eigene Burn-out-Risiko.

Die langfristigen Konsequenzen der Pandemie auf die psychische Gesundheit von Kindern und Jugendlichen sind noch nicht vollständig absehbar und sollten in ihrer Erforschung Priorität erfahren (Holmes et al. 2020). Zukünftige Studien sollten zusätzlich das Befinden der behandelnden Kinder- und JugendlichenpsychotherapeutInnen in den Blick nehmen. Daraus sollten Möglichkeiten zur Verbesserung der therapeutischen Arbeitssituation abgeleitet werden, um eine langfristige psychotherapeutische Versorgung von Kindern und Jugendlichen zu gewährleisten.

## Fazit für die Praxis

- Die COVID-19-Pandemie hat weitreichende Auswirkungen auf die ambulante psychotherapeutische Versorgung von Kindern und Jugendlichen in Deutschland.
- Die Wartezeiten auf einen Versorgungsplatz haben sich nahezu verdoppelt, obwohl die Mehrzahl der TherapeutInnen ihre Behandlungsstunden erhöht hat. Bei der Hälfte der PatientInnen hat sich die Symptomatik verschlechtert. Psychische Störungen treten seit Pandemiebeginn häufiger auf; die interdisziplinäre Zusammenarbeit ist erschwert. TherapeutInnen scheinen sich selbst durch die Pandemie belastet zu fühlen. Vereinzelt wird eine wachsende Sorge um das eigene Burn-out-Risiko berichtet.
- In der aktuellen Krisensituation ist das Versorgungssystem an den gestiegenen Bedarf anzupassen. Zukünftig bedarf es der regelmäßigen Kontrolle des Verhältnisses von Therapiebedarf und -angebot.
- Studien sollten das Befinden der behandelnden Kinder- und JugendlichenpsychotherapeutInnen in den Blick nehmen, um Verbesserungsmöglichkeiten der therapeutischen Arbeitssituation ableiten zu können.

## Supplementary Information




