# Supplementary material for: Impact of the COVID-19 pandemic on outpatient psychotherapy for children and adolescents
Source: Psychotherapie. 2022 Jun 27;67(6):469–77. [Article in German] doi: 10.1007/s00278-022-00604-y (PMC9243913; doi:10.1007/s00278-022-00604-y)
Supplement: Supplementary file 1 [file 278_2022_604_MOESM1_ESM.pdf]

## Fragebogen

### 1 Demografische Daten

**Wie alt sind Sie?**

Bitte tragen Sie ihr derzeitiges Alter in ganzen Zahlen ein:

[ ] Jahre

**Welchem Geschlecht fühlen Sie sich zugehörig?**

Bitte klicken Sie eine der drei Optionen an.

- [ ] weiblich
- [ ] männlich
- [ ] divers

**In welchem Bundesland arbeiten Sie?**

Bitte klicken Sie eine der nachfolgenden Optionen an.

- [ ] Baden-Württemberg
- [ ] Nordrhein-Westfalen
- [ ] Bayern
- [ ] Saarland
- [ ] Hessen
- [ ] Schleswig-Holstein
- [ ] Niedersachsen
- [ ] Rheinland-Pfalz
- [ ] Hamburg
- [ ] Bremen
- [ ] Thüringen
- [ ] Sachsen-Anhalt
- [ ] Sachsen
- [ ] Brandenburg
- [ ] Mecklenburg-Vorpommern
- [ ] Berlin

**In welchem Siedlungsgebiet arbeiten Sie?**

Bitte klicken Sie eine der nachfolgenden Optionen an.

- [ ] im ländlichen Raum (bis 4.999 Einwohner)
- [ ] in einer kleinen oder mittleren Stadt (5.000 - 99.000 Einwohner)
- [ ] in einer kleineren Großstadt (100.000 - 499.000 Einwohner)
- [ ] in einer großen Großstadt (ab 500.000 Einwohner)

### 2 Therapeutische Arbeitssituation

**Wie lange arbeiten Sie als Psychotherapeut*in mit Kindern und Jugendlichen?**

Bitte tragen Sie die Arbeitszeit in ganzen Jahren ein.

[ ] Jahre

**Wo arbeiten Sie?**

Bitte klicken Sie eine der beiden nachfolgenden Optionen an.

- ○ in einer vertragspsychotherapeutischen Praxis (Kassensitz) oder
- ○ in einer Privatpraxis oder
- ○ in einer anderen Institution der ambulanten psychotherapeutischen Versorgung (z. B. Hochschulambulanz, MVZ)?

**Arbeiten Sie:**

- ○ mit einer Einzelapprobation als Kinder- und Jugendlichenpsychotherapeut*in oder
- ○ mit einer Doppelapprobation als Psychologische Psychotherapeut*in und Kinder- und Jugendlichenpsychotherapeut*in oder
- ○ als psychologische/r Psychotherapeut*in mit Abrechnungsgenehmigung für Kinder und Jugendliche ?

**Arbeiten Sie:**

- ○ mit einer Teilzulassung (halbem Kassensitz) oder
- ○ mit einer vollen Zulassung (ganzer Kassensitz) oder
- ○ als Angestellte/r ?

## 3 Veränderung der Anfragen

**Bitte schätzen Sie, wie viele der aktuellen Neuanfragen bezüglich eines Erstgesprächs/Therapieplatzes von Ihnen schon einmal behandelte Patient*innen waren.**

Bewegen Sie bitte den Schieberegler auf die geschätzte Prozentzahl (Ø letzte 6 Monate).

**Bitte schätzen Sie, wie viele der Neuanfragen bezügliches eines Erstgesprächs/Therapieplatzes vor 2 Jahren von Ihnen schon einmal behandelte Patient*innen waren.**

Bewegen Sie bitte den Schieberegler auf die geschätzte Prozentzahl (Ø vor 2 Jahren).

**Bitte schätzen Sie, wie viele Wochen ein Kind/Jugendlicher derzeit auf einen Erstgesprächstermin bei Ihnen wartet.**

Tragen Sie bitte die durchschnittliche Wartezeit (**Ø** letzte **6 Monate)** in Wochen und ganzen Zahlen ein.

[ ] Wochen

**Bitte schätzen Sie, wie viele Wochen ein Kind/Jugendlicher vor 2 Jahren durchschnittlich auf ein Erstgespräch bei Ihnen wartete.**

Tragen Sie bitte die durchschnittliche Wartezeit (vor 2 Jahren) in Wochen und ganzen Zahlen ein.

[ ] Wochen

**Bitte schätzen Sie, wie viele Wochen ein Kind/Jugendlicher derzeit auf einen Therapieplatz bei Ihnen wartet.**

Tragen Sie bitte die durchschnittliche Wartezeit (Ø letzte 6 Monate) in Wochen und ganzen Zahlen ein.

[ ] Wochen

**Bitte schätzen Sie, wie viele Wochen ein Kind/Jugendlicher durchschnittlich vor 2 Jahren auf einen Therapieplatz bei Ihnen wartete.**

Tragen Sie bitte die durchschnittliche Wartezeit (vor 2 Jahren) in Wochen und ganzen Zahlen ein.

[ ] Wochen

## 4 Veränderung der Therapieangebote

**Bitte schätzen Sie, wie sich nachfolgende psychotherapeutische Leistungen in den letzten 6 Monaten im Vergleich zur Vor-Pandemie-Situation vor 2 Jahren bei Ihnen verändert haben.**

Bitte klicken Sie an, wie sich die jeweilige Leistung verändert hat.

| | sehr stark verringert | stark verringert | leicht verringert | gleich | leicht erhöht | stark erhöht | sehr stark erhöht |
|---|---|---|---|---|---|---|---|
| ein Erstgespräch/eine Sprechstunde durchgeführt | ○ | ○ | ○ | ○ | ○ | ○ | ○ |
| probatorische Sitzungen durchgeführt | ○ | ○ | ○ | ○ | ○ | ○ | ○ |

| | | | | | | | |
|---|---|---|---|---|---|---|---|
| eine Kurzzeittherapie nach Probatorik begonnen | ○ | ○ | ○ | ○ | ○ | ○ | ○ |
| eine Langzeittherapie nach Probatorik begonnen | ○ | ○ | ○ | ○ | ○ | ○ | ○ |
| eine Akuttherapie nach Probatorik begonnen | ○ | ○ | ○ | ○ | ○ | ○ | ○ |
| eine psychotherapeutische Behandlung mit einem Kind/Jugendlichen verlängert | ○ | ○ | ○ | ○ | ○ | ○ | ○ |
| eine psychotherapeutische Behandlung mit einem Kind/Jugendlichen ausgesetzt | ○ | ○ | ○ | ○ | ○ | ○ | ○ |
| eine psychotherapeutische Behandlung mit einem Kind/Jugendlichen abgebrochen | ○ | ○ | ○ | ○ | ○ | ○ | ○ |

## 5 Veränderung der Therapieformate

**Bitte schätzen Sie, wie sich Ihre Behandlungsformate verändert haben.**

Bitte klicken Sie an, wie sich das jeweilige Behandlungsformat in den letzten 6 Monaten im Vergleich zur Vor-Pandemie-Situation vor 2 Jahren verändert hat.

| | sehr stark verringert | stark verringert | leicht verringert | gleich | leicht erhöht | stark erhöht | sehr stark erhöht |
|---|---|---|---|---|---|---|---|
| Präsenzbehandlungen mit Kindern/Jugendlchen | ○ | ○ | ○ | ○ | ○ | ○ | ○ |
| Präsenzbehandlungen mit einer Bezugsperson | ○ | ○ | ○ | ○ | ○ | ○ | ○ |
| Präsenz-Gruppenbehandlungen mit Kindern/Jugendlichen | ○ | ○ | ○ | ○ | ○ | ○ | ○ |
| Präsenz-Gruppenbehandlungen mit Bezugspersonen | ○ | ○ | ○ | ○ | ○ | ○ | ○ |
| telefonische Konsultationen mit Kindern/Jugendlichen | ○ | ○ | ○ | ○ | ○ | ○ | ○ |
| telefonische Konsultationen mit Bezugspersonen | ○ | ○ | ○ | ○ | ○ | ○ | ○ |
| Video-Behandlungen mit Kindern/Jugendlichen | ○ | ○ | ○ | ○ | ○ | ○ | ○ |
| Video-Behandlungen mit Bezugspersonen | ○ | ○ | ○ | ○ | ○ | ○ | ○ |

## 6 Verschlechterung der Symptome

**Bitte schätzen Sie, wie hoch der Anteil von Ihnen behandelter Kinder/Jugendlicher in den letzten 6 Monaten war, bei denen eine pandemieassoziierte Verschlechterung bezüglich Symptomanzahl/-schwere festgestellt wurde.**

**Bewegen Sie bitte den Schieberegler auf die geschätzte Prozentzahl.**

**Was waren aus Ihrer Sicht Gründe für die Verschlechterung der Symptomanzahl/-schwere?**

Beim Anklicken sind Mehrfachnennungen möglich.

- ☐ Wegfall/Veränderung der Tagesstruktur
- ☐ konkrete Angst vor Coronainfektion
- ☐ allgemeine Unsicherheit/Bedrohlichkeit/Unvorhersehbarkeit
- ☐ eingeschränkte soziale Kontakte
- ☐ eingeschränktes Freizeitangebot
- ☐ Ausgangssperren/Radiusbeschränkungen
- ☐ finanzielle Schwierigkeiten
- ☐ lange Wartezeit auf Therapieplatz
- ☐ vermehrte Konflikte im Elternhaus
- ☐ Überlastung/Überforderung der Eltern
- ☐ Home schooling/Wechselunterricht
- ☐ Wegfall/Reduktion von Angeboten des Hilfesystems
- ☐ Gewalt in der Familie
- ☐ Psychische Erkrankung der Eltern
- ☐ Psychische Erkrankung von Geschwistern
- ☐ andere Gründe ______

## 7 Veränderungen Störungsbilder

**Bitte schätzen Sie, wie sich die nachfolgenden diagnostizierten Störungsbilder bei Ihnen in den letzten 6 Monaten im Vergleich zur Vor-Pandemie-Situation vor 2 Jahren verändert haben.**

Bitte klicken Sie die Veränderung der jeweiligen Diagnosestellung an.

| | sehr viel seltener | viel seltener | seltener | gleich | häufiger | viel häufiger | sehr viel häufiger |
|---|---|---|---|---|---|---|---|
| Depressionen | ○ | ○ | ○ | ○ | ○ | ○ | ○ |
| Angststörungen | ○ | ○ | ○ | ○ | ○ | ○ | ○ |
| Hyperkinetische Störungen | ○ | ○ | ○ | ○ | ○ | ○ | ○ |
| Störungen des Sozialverhaltens | ○ | ○ | ○ | ○ | ○ | ○ | ○ |
| Essstörungen | ○ | ○ | ○ | ○ | ○ | ○ | ○ |
| Schlafstörungen | ○ | ○ | ○ | ○ | ○ | ○ | ○ |
| Zwangsstörungen | ○ | ○ | ○ | ○ | ○ | ○ | ○ |
| Akute und Posttraumatische Belastungsstörungen | ○ | ○ | ○ | ○ | ○ | ○ | ○ |
| Anpassungsstörungen | ○ | ○ | ○ | ○ | ○ | ○ | ○ |

| | | | | | | | |
|---|---|---|---|---|---|---|---|
| frühkindliche Regulationsstörungen | ○ | ○ | ○ | ○ | ○ | ○ | ○ |
| Psychische und Verhaltensstörungen durch psychotrope Substanzen | ○ | ○ | ○ | ○ | ○ | ○ | ○ |
| Abhängiger Mediengebrauch (PC, TV, Handy etc.) | ○ | ○ | ○ | ○ | ○ | ○ | ○ |
| Schulabsentismus | ○ | ○ | ○ | ○ | ○ | ○ | ○ |
| bisher ungenanntes Störungsbild, was sich veränderte | ○ | ○ | ○ | ○ | ○ | ○ | ○ |

## 8 Zusammenarbeit interdisziplinär

**Bitte schätzen Sie die Zusammenarbeit im interdisziplinären Netzwerk in den letzten 6 Monaten im Vergleich zur Vor-Corona-Pandemie-Situation vor 2 Jahren ein.**

Bitte klicken Sie an, wie sich die jeweilige Zusammenarbeit verändert hat.

| | sehr stark verringert | stark verringert | leicht verringert | gleich | leicht erhöht | stark erhöht | sehr stark erhöht |
|---|---|---|---|---|---|---|---|
| mit anderen niedergelassenen Therapeut*innen (z.B. Austausch, Intervision, Supervision) | ○ | ○ | ○ | ○ | ○ | ○ | ○ |
| mit Kliniken für Kinder- und Jugendpsychiatrie/Psychosomatik | ○ | ○ | ○ | ○ | ○ | ○ | ○ |
| mit externen Therapeut*innen (Logo-/Ergo-/Physio-/Lerntherapeuten etc.) | ○ | ○ | ○ | ○ | ○ | ○ | ○ |
| mit Pädagog*innen (Lehrer*innen, Erzieher*innen, Sozialpädagog*innen) | ○ | ○ | ○ | ○ | ○ | ○ | ○ |
| mit der Jugendhilfe | ○ | ○ | ○ | ○ | ○ | ○ | ○ |
| mit den Eltern/-teilen/ Bezugspersonen | ○ | ○ | ○ | ○ | ○ | ○ | ○ |

## 9 persönliche Situation Therapeut*innen

**Bitte schätzen Sie, wie sich die Anzahl der von Ihnen angebotenen Behandlungsstunden in den letzten 6 Monaten im Vergleich zur Vor-Pandemie-Situation vor 2 Jahren verändert hat.**

| | sehr stark verringert | stark verringert | leicht verringert | gleich | leicht erhöht | stark erhöht | sehr stark erhöht |
|---|---|---|---|---|---|---|---|
| angebotene Behandlungsstunden | ○ | ○ | ○ | ○ | ○ | ○ | ○ |

**Bitte schätzen Sie, in welchem Maß sich die nachfolgenden Faktoren auf die angebotenen Behandlungsstunden auswirkten.**

Klicken Sie bitte an, in welchem Maß die einzelnen Faktoren die Anzahl der von Ihnen angebotenen Behandlungsstunden in den letzten 6 Monaten beeinflussten.

| | sehr häufig | häufig | selten | sehr selten | gar nicht |
|---|---|---|---|---|---|
| Infektionsschutzmaßnahmen wie Lüften, Waschen etc. | ○ | ○ | ○ | ○ | ○ |
| Home schooling/Wechselunterricht | ○ | ○ | ○ | ○ | ○ |

| | | | | | |
|---|---|---|---|---|---|
| der eigenen Kinder | | | | | |
| Care-Arbeit (Kinderbetreuung, ältere Angehörige, vermehrte Hausarbeit) | ○ | ○ | ○ | ○ | ○ |
| eigene Infektionsängste | ○ | ○ | ○ | ○ | ○ |
| erhöhter Aufwand durch neue Therapieformate | ○ | ○ | ○ | ○ | ○ |
| Wegfall Gruppentherapien | ○ | ○ | ○ | ○ | ○ |
| behördlich verordnete häusliche Quarantäne | ○ | ○ | ○ | ○ | ○ |
| erhöhtes Anfrageaufkommen | ○ | ○ | ○ | ○ | ○ |
| anderer Grund: | ○ | ○ | ○ | ○ | ○ |

## 10 Unerwartetes/Ungenanntes

**Welche unerwarteten bzw. bisher ungenannten Veränderungen haben Sie seit Beginn der Corona-Pandemie als Psychotherapeut*in mit Kindern/Jugendlichen wahrgenommen?**

Bitte tragen Sie ein, was Ihnen in den letzten 6 Monaten im Vergleich zur Vor-Pandemie-Situation vor 2 Jahren aufgefallen ist. Dies betrifft sowohl Sie als Therapeut*in als auch ihre Patient*innen und deren Bezugspersonen sowie das interdisziplinäre Netzwerk.

## 11 Endseite

Wir danken Ihnen für die Teilnahme an dieser Studie und würden uns freuen, wenn Sie den Link der Studie an Kolleg*innen weiterleiten.
Mit besten Grüßen aus Leipzig und Koblenz-Landau
